# Influence of optical aberrations on depth-specific spatial frequency domain techniques

**DOI:** 10.1117/1.JBO.27.11.116003

**Published:** 2022-11-10

**Authors:** Motasam Majedy, Nandan K. Das, Johannes Johansson, Rolf B. Saager

**Affiliations:** Linköping University, Department of Biomedical Engineering, Linköping, Sweden

**Keywords:** spatial frequency domain imaging, aberrations, subsurface optical properties, quantitative spectroscopy, tissue simulating phantoms

## Abstract

**Significance:**

Spatial frequency domain imaging (SFDI) and spatial frequency domain spectroscopy (SFDS) are emerging tools to non-invasively assess tissues. However, the presence of aberrations can complicate processing and interpretation.

**Aim:**

This study develops a method to characterize optical aberrations when performing SFDI/S measurements. Additionally, we propose a post-processing method to compensate for these aberrations and recover arbitrary subsurface optical properties.

**Approach:**

Using a custom SFDS system, we extract absorption and scattering coefficients from a reference phantom at 0 to 15 mm distances from the ideal focus. In post-processing, we characterize aberrations in terms of errors in absorption and scattering relative to the expected in-focus values. We subsequently evaluate a compensation approach in multi-distance measurements of phantoms with different optical properties and in multi-layer phantom constructs to mimic subsurface targets.

**Results:**

Characterizing depth-specific aberrations revealed a strong power law such as wavelength dependence from ∼40 to ∼10% error in both scattering and absorption. When applying the compensation method, scattering remained within 1.3% (root-mean-square) of the ideal values, independent of depth or top layer thickness, and absorption remained within 3.8%.

**Conclusions:**

We have developed a protocol that allows for instrument-specific characterization and compensation for the effects of defocus and chromatic aberrations on spatial frequency domain measurements.

## 1 Introduction

Spatial frequency domain techniques use spatially modulated illumination patterns to quantify absorption and scattering properties of tissue and other diffusive media across visible and/or near-infrared ranges.^1^^–^^4^ These techniques can be implemented in a variety of ways such as spatial frequency domain imaging (SFDI)^2^ or spatial frequency domain spectroscopy (SFDS).^3^ Through the quantification of these optical properties, SFDI/S has demonstrated the potential to non-invasively assess skin cancers,^5^^,^^6^ wound severity,^7^^,^^8^ and tissue structure and viability^9^^,^^10^ in the skin. In many of these clinical applications, such as burn wound assessment and melanin quantification, depth sensitivity and subsurface discrimination approaches enabled by SFDI/S are critical features toward accurate characterization of specific tissue volumes.^11^^–^^15^

Spatial frequency domain techniques utilize a light transport model-based approach to relate the measured remitted reflectance from turbid media, such as tissue, to quantitative values of absorption and reduced scattering. These models presume a uniform distribution of light upon which the spatial frequencies are encoded, and the propagation of this planar illumination pattern will only be impacted by the absorption and scattering properties within the tissue volume. To that end, the optical characteristics of the instrument must be accounted for to isolate measured reflectance signals resulting from the interaction of these illumination patterns with the tissue itself. This task is typically performed in a calibration procedure where a fully characterized reference phantom is measured either prior to or after the target medium.^2^^,^^3^ These reference phantoms play a critical role, as their absorption and scattering properties are known in advance and hence can characterize these instrumentation contributions to the detected reflectance signals.

Though not often acknowledged explicitly in the literature, this calibration approach using turbid reflectance standards not only will correct for inhomogeneities in illumination intensity over the field of view at each individual spatial frequency, but also any out-of-plane degradation of the pattern integrity (independent from the tissue’s intrinsic optical properties) as the planar illumination pattern propagates into the tissue volume. These out-of-plane degradations are a result of the optical design of the SFDI/S system, primarily its depth of focus and chromatic aberrations, which change as a function of distance relative to the ideal image plane. Since many SFDI/S systems are built around pre-existing projection units, the optical design is often embedded with the digital micromirror device and optimized for wide-field projection applications in either the visible or near-infrared wavelength regimes.^16^^–^^20^ This has led to systems that reduce the projection field of view with additional lenses. This type of modification also reduces the working distance between the instrument and the target medium, which has advantages toward the development of compact handheld systems. This reduced working distance, however, can also reduce the depth of focus. This could rapidly blur the projected pattern as it propagates into the tissue volume and compete with the effects of light scattering. Likewise, several SFDI/S approaches span both visible- and near-infrared regimes,^3^^,^^6^^,^^8^^,^^13^^,^^17^ which also can be complicated by chromatic aberrations as most achromatic designs only compensate for this type of aberration over a few hundred nanometers.

It has been shown that when the target tissue has a distinct surface shape and depth (i.e., topology), errors in the determination of optical properties may occur due to the tissue surface’s distance relative to the ideal imaging plane of the SFDI/S instrument.^21^ Previous studies have employed sample surface profile information to adjust for venous imaging^22^ or imitate diffuse reflectance of biological tissues.^23^ Similar approaches, combined with multi-step calibrations procedures, have also been developed to correct for this displacement error in the context of SFDI/S.^13^^,^^24^

In this work, we will present a simple method using a single tissue-simulating phantom to evaluate the influence of out-of-plane optical aberrations in the context of the resulting absorption and reduced scattering coefficients. As different spatial frequency instrument designs will exhibit their own distinct combinations and magnitudes of optical aberrations, this characterization method can provide vital feedback on the accuracy and spectral integrity of depth-specific optical properties. Additionally, once this optical characterization is obtained, we will also demonstrate how this data can be used to compensate for these aberration errors in post-processing for a spatial frequency domain setup that is particularly susceptible to these types of aberrations (e.g., a compact SFDS system). This compensation approach is evaluated both in measurements of a phantom with differing optical properties of that used in characterization and in multi-layer phantom constructs. The former one demonstrates that this empirical method can be applied to arbitrary tissue optical properties over a range of positional displacements from the ideal image plane and in the latter case demonstrates that sub-surface optical properties can be extracted when the top layer properties are known.

## 2 Material and Method

### 2.1 SFDS Instrument under Investigation

The measurement technique in this configuration uses spatially modulated sinusoidal light patterns of visible- and near-infrared light projected at varying spatial frequencies, which are collected from a single point as delivered to a spectrometer via an optical fiber. This specific SFDS instrument was chosen for two reasons: (1) its compact optical design and short working distance makes it susceptible defocus errors and (2) spanning visible and near-infrared regimes makes it susceptible to chromatic aberrations. The data collection and processing of SFDS data have been explained in detail elsewhere.^2^^,^^3^ To project structured light patterns with five equally spaced spatial frequencies ranging from 0 to $0.2\;{\mathrm{mm}}^{-1}$ this instrument employs a 150-W quartz-tungsten-halogen light source (21DC-3AHD-TQB-FILT, Techniquip) coupled to a digital micromirror device, DMD, (AJP-4500 DMD Projector, Ajile Light Industries Inc.) via a fiber optic bundle.

This projection unit was modified through the addition of a 50-mm focal length lens at the distal end of its integrated lens system, resulting in a 55×37  mm field of view at a working distance of 75 mm. The light was collected by the optical fiber and delivered to a spectrometer (AvaSpec-ULS2048CL-EVO-VA-50, Avantes BV). In the context of this paper, we chose to use a range of 432 to 950 nm due to the limited signal-to-noise ratio (SNR) for the extreme wavelengths.

To reject specular reflection from the sample's surface, polarizing filters were used in the system. The first polarizer was positioned after the DMD, and the analyzer was inserted between the collection lens and the fiber optic. To improve the SNR, an average of 40 measurements were used for each spatial frequency.

### 2.2 Experimental Setup and Data Collection

Phantoms are frequently used to test a system's or algorithm’s accuracy. A step-by-step process for fabricating silicone phantoms adjusted to certain optical properties in the visible and near-infrared range is detailed elsewhere.^25^ In this investigation, we used a single reference phantom to characterize the depth-specific errors the instruments optical aberrations impart. For this investigation, it will be referred to as the “reference phantom”. This phantom used ${\mathrm{TiO}}_{2}$ [Titanium (IV) Oxide, anatase, Sigma-Aldrich] as the scattering agent, and the absorber was India ink (Pebeo). The optical properties of the phantom were independently characterized using multiple optical techniques, including inverse adding doubling, multi-distance spectroscopy, and SFDI.^26^ A second homogenous phantom was used to evaluate how well this method could be applied to turbid media of arbitrary optical properties over a range of positional displacements. This phantom will be referred to as the “test phantom.” Red food dye (Dr. Oetker) was used as an absorber as it loosely mimics hemoglobin absorption but also provides variation in wavelength-specific absorption unlike the relatively flat absorption from India ink. In this test phantom, ${\mathrm{TiO}}_{2}$ was used as a scattering agent. Lastly, to mimic subsurface targets, three thin phantoms containing the same concentration of India ink and ${\mathrm{TiO}}_{2}$ were placed in direct contact on top of the test phantom, which serves as the bottom layer. These three phantoms had thicknesses of 1.93, 2.58, and 3.19 mm, and absorption and reduced scattering coefficients of 0.015 and $0.49\;{\mathrm{mm}}^{-1}$ at 650nm. This set of phantoms will be referred to as “layered phantoms.”

Both the reference and test phantoms were measured using the SFDS system at 11 distances from the ideal image place (referred to as “in-focus”). These distances were each incremented by 1 mm up to 10 mm. An additional measurement at 15 mm was also collected to provide data at a displacement beyond what one would expect to measure by SFDI/S techniques. The motivation behind this extreme data point is to evaluate whether this correction method remains valid well beyond typical measurement parameters. For the multi-layer phantom constructs, three measurements were collected where each top layer thickness phantom was positioned at the in-focus plan, resulting in the bottom layer phantom being displaced by the thickness of the top layer.

All measurements were processed using a standard homogeneous Monte Carlo model for SFDI data.^2^^,^^3^ For all these datasets, a single reference calibration, acquired in focus, was used. From these process results, the determined absorption and reduced scattering coefficients were then analyzed independently.

### 2.3 Characterization Method

#### 2.2.1 Interpretation and modeling of aberration influence on the scattering coefficient

From a light transport model perspective in the spatial frequency domain, scattering essentially blurs out the contrast of the projected pattern as it propagates through turbid media. Defocus aberrations will also reduce the contrast of these spatial frequency encoded patterns, whereas chromatic aberrations will displace where in depth the ideal image place is formed as a function of wavelength. We assert that these mechanisms, however, are independent from scattering as aberrations will only propagate in unscattered (and unabsorbed) light.

To characterize the impact of depth-dependent aberrations, the ratios of each reduced scattering coefficient spectra at all depths were calculated relative to the phantom in the focused position. These scaling errors were then fitted to an empirical algebraic equation. This was done to remove any measurement noise and establish a smooth mesh that describes the non-linear influence these aberrations impart. Though arbitrarily derived, Eq. (1) was used for modeling these scaling errors, α(λ,z), as it provided the most robust fitting across the full spectral range at all depths measured. As the deviations in scattering due to optical aberrations are described in fractional units, the resulting look-up table (LUT) is designed to be applied to any arbitrary scattering spectrum,

$$\alpha (\lambda ,z)=-c_{1}\times \lambda^{-c_{2}(z)}+c_{3}\times \lambda +c_{4}(z).$$ (1)

#### 2.2.2 Interpretation and modeling of the absorption coefficient

Unlike scattering, the absorption coefficient does not directly compete with optical aberrations. Absorption would not alter the photon’s trajectory, rather it terminates its further propagation. For that reason, the influence of aberrations on the determination of the absorption coefficient will be more complex and indirect. As typically described in terms of Beer’s law, $I(l_{p})=I_{0\;}e^{-\mu_{a}l_{p}}$, there are two key components to keep in mind when considering how aberrations could distort absorption values. The first of these is the source intensity, $I_{0}$. Here, it is presumed that as light propagates through non-scattering media, the reduction of light intensity is due to losses from absorption. However, the aberrations we are considering in this investigation will also reduce the light intensity as the field of view of the projected pattern will expand slightly as it propagates in depth. We chose to model this additional source of error as a subtractive offset in the resulting amplitude of the depth-specific absorption value. Second, the effective pathlength, $l_{p}$, that photons travel in turbid media is now not just dependent on the scattering properties, but rather, the combination of scattering and aberration errors. For this second component, we choose to apply a scaling factor, α(λ,z), utilizing the same characterization model.

For this characterization procedure, two steps were applied. First, the depth-specific absorption spectra from the reference phantom were normalized to the in-focus measurement at an arbitrary wavelength. This normalization established the linear offset of each displaced absorption coefficient as a function of depth due to the defocusing reduction of light intensity. For this investigation, we chose 650 nm. Once this normalization was performed, the scaling error LUT was calculated for all wavelengths, at all depths, and the coefficients (c1,c2,c3,c4) were obtained by fitting it to the same mathematical model.

### 2.4 Compensation Method

#### 2.4.1 Evaluation of displaced homogeneous phantom

For the test phantom, the reduced scattering coefficients at different respective depths were compensated by dividing the scattering spectrum by the interpolated, depth-specific scaling factors from the LUT designed from previous section. As for the absorption, the resulting depth-specific coefficients from the LUT multiplied to absorption spectrum first and then the depth-specific absorption spectra were subtracted by the linear offset coefficient that accounts for the scalar reduction of light intensity, $I_{0}$.

#### 2.4.2 Evaluation of multi-layered constructs

For the multi-layer constructs as mentioned before the top layer thickness phantom with known optical properties was placed at the in focused position, resulting the bottom layer to be displaced by the thickness of the top layer. Utilizing a two-layer model proposed in previous studies, the top layer properties were accounted for in the context of their relative, wavelength-specific partial volume of the total estimated depth of interrogation.^11^^,^^27^^,^^28^ The same compensation procedure was then applied to the extracted bottom layer optical properties to illustrate that optical aberrations are independent of optical properties of the phantom, even in multi-layer structures.

## 3 Results

**Fig. 1:**
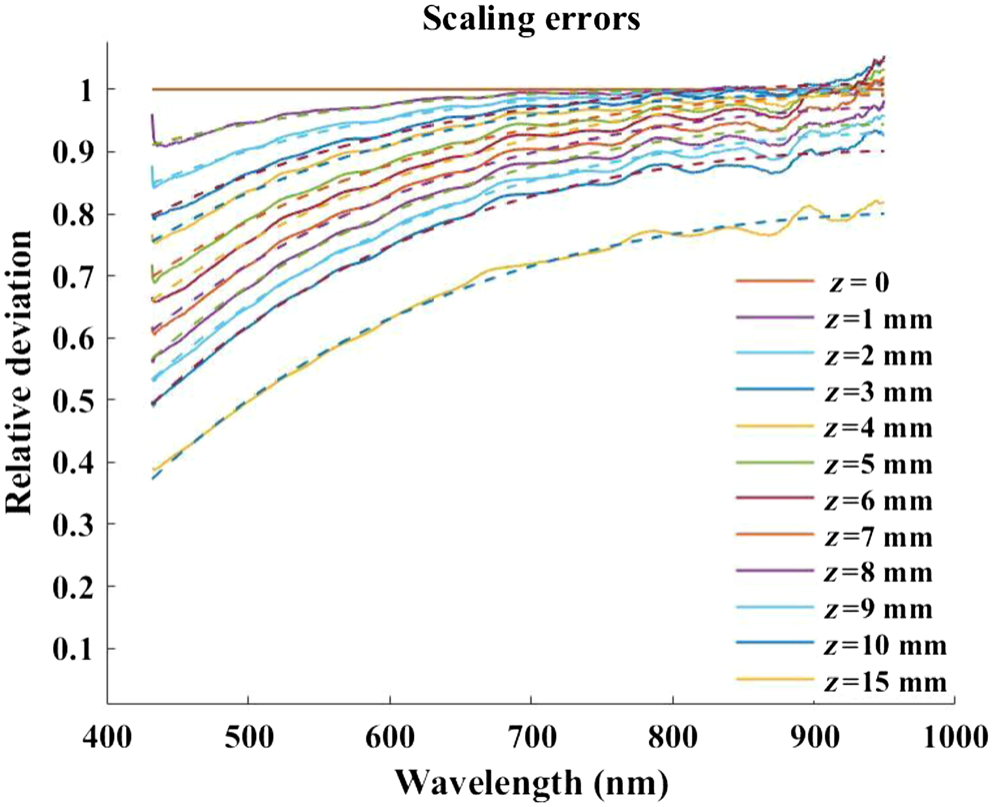
The calculated ratios of each reduced scattering coefficient spectra at all depths relative to when the phantom was in the focused position (z=0), dashed lines are the data fitted using Eq. (1).

The scaling errors from measured data relative to when the phantom was placed in the focused position are shown in Fig. 1 with solid lines. Here, there is a progressive underestimation of scattering properties as a function of increasing distance from the focal plane. The dashed lines show the fit curves [Eq. (1)] across the full spectral range at all depths. These fittings were done to remove the measurement noise artifacts and establish a smooth mesh that can describe the non-linear influence that the aberrations convey on the resulting reduced scattering coefficient. An LUT was generated from these fitted curves that could be interpolated to any arbitrary depth up to 15 mm. To compensate for aberrations, measured scattering values would be divided by the interpolated depth-specific curve from this table, absorption values would be multiplied by it. Additionally, the offset term for the absorption values was determined to be $0.00025\;{\mathrm{mm}}^{-1}$ per millimeter depth for this specific instrument setup. This offset value was highly linear over this 15-mm range, with $R^{2}=0.999$.

**Fig. 2:**
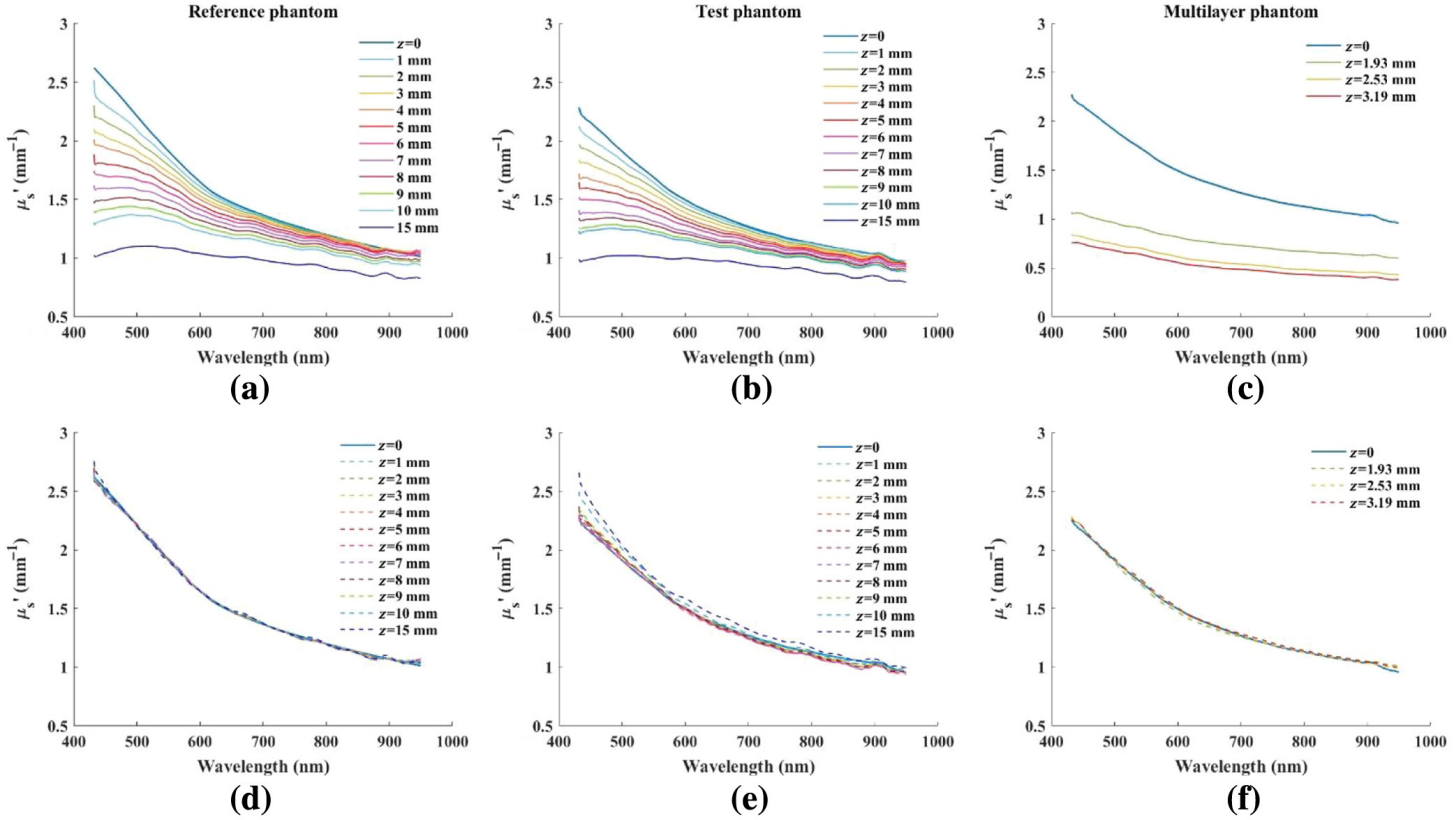
Reduced scattering coefficient calculated at each distance and the respective aberration compensated data for the (a) and (d) reference phantom; (b) and (e) test phantom; and (c) and (f) multi-layered phantoms. z=0 refers to the ideal, in-focus reduced scattering spectra.

The measured reduced scattering spectra for the reference phantom, test phantom, and the multi-layered constructs at different distances to the projection lens is shown in Figs. 2(a)–2(c). As shown, the reduced scattering coefficient at different distances from the projection lens progressively deviates from the in-focus measurement. The compensated reduced scattering spectra are shown in Figs. 2(d)–2(f) for the reference phantom, the test phantom, and the multi-layered construct, respectively. These spectra were corrected using the characterization data obtained from reference phantom only. After applying the aberration compensation method, the scattering spectra of the reference phantom from all distances are within an average of 0.1% root-mean-square (RMS) error relative to the in-focus values (0.2% at 10 mm from the focal plane). Left uncorrected, these RMS errors would be 18.2% on average and 40.2% at 10 mm. The average for the test phantom scattering was 1.4% over all distances (uncorrected average: 17.1%, 36% at 10 mm) and ∼0.6% for the multi-layered construct.

**Fig. 3:**
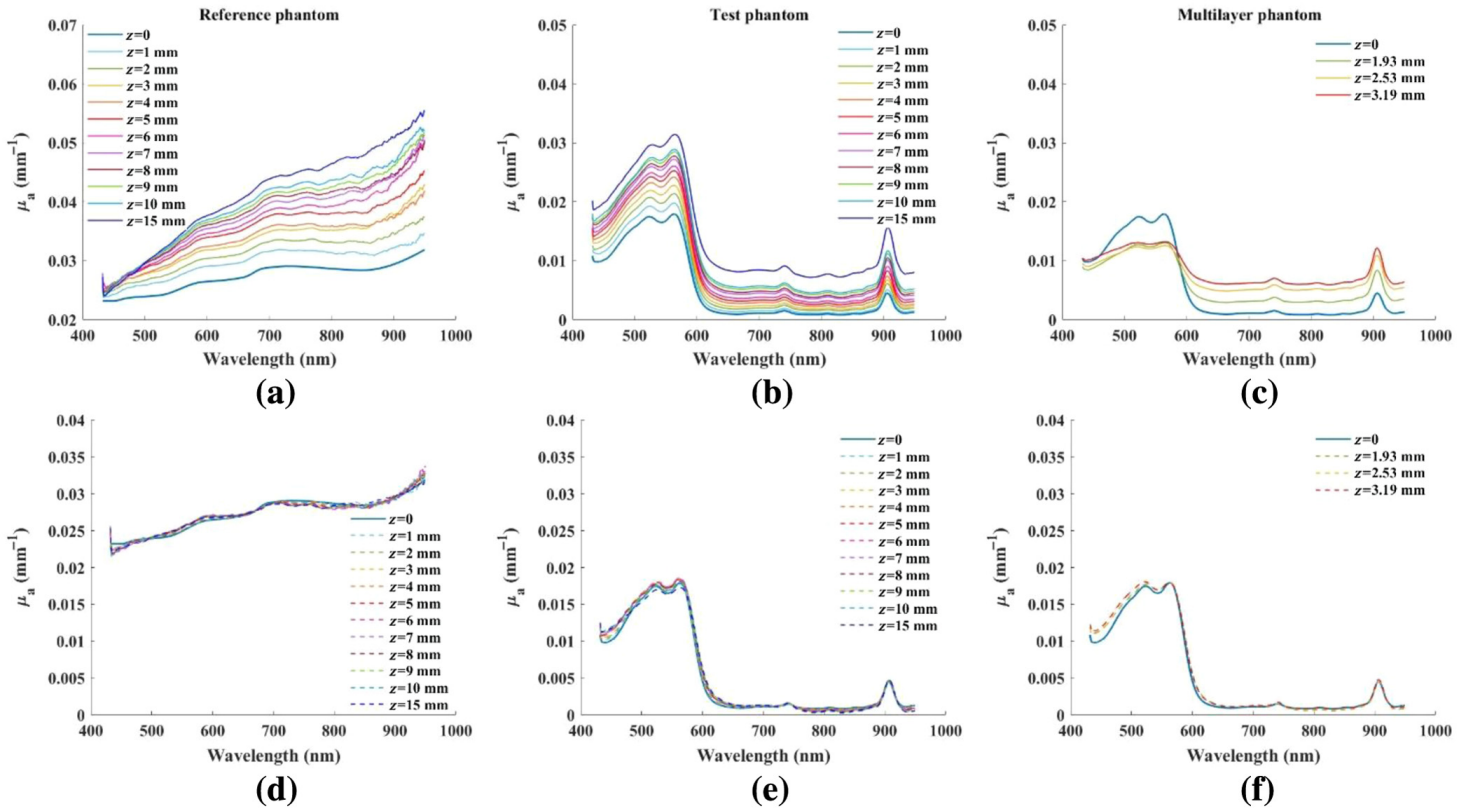
Absorption coefficient calculated at each distance and the respective recovered aberration compensated data for the (a) and (d) reference phantom; (b) and (e) test phantom; and (c) and (f) multi-layered phantoms. z=0 refers to the ideal, in-focus absorption spectra.

The measured absorption spectra from the reference, test and multi-layer phantoms are shown in Figs. 3(a)–3(c), respectively. Figures 3(d)–3(f) shows the recovered optical properties for absorption coefficient after correcting for the errors, using the characterization data obtained from reference phantom only. There is on average a ∼32.2% RMS error in uncorrected absorption spectra relative to the in-focus values for the reference phantom (45.5% at 10 mm). After applying the compensation method, the absorption RMS error reduced to within 0.03% on average. The test phantom showed an average ∼49.1% RMS error (69.5% at 10 mm) that was reduced to an average of 3.8% over all distances (3.4% at 10 mm) and for the multi-layered construct’s average of ∼43.7% was reduced to 4.7%.

Overall, the data shows that the recovery of both out-of-focus and subsurface optical properties can be achieved even though these optical aberrations can have a considerable impact on the directly measured spatial frequency dependent reflectance values.

## 4 Discussion

We have presented a method to characterize and subsequently compensate for the depth-specific impact that optical aberrations can have in spatial frequency domain measurements. The goal of this investigation was to demonstrate that while these aberrations can significantly impact the determination and spectral integrity of subsurface optical properties, they can be treated independent from the intrinsic values of the turbid medium. Using a test phantom different from that used to characterize the systems aberrations, we have shown that the compensation method can be applied as a scaling factor (and an additional offset, in the case of weak absorption) irrespective of the target's optical properties, reducing errors on average by a factor of ∼12. While not typical in most practical settings, this compensation approach was shown to remain effective even at such extremes as 15-mm displacement errors. Additionally, to further illustrate this independence of aberrations from the target’s properties, multi-layer phantoms were used where the bottom layer optical properties were recovered to within <5% its target.

This approach does not directly address the effect of aberrations on the spatial frequency dependent reflectance, but rather on its resulting estimation of absorption and reduced scattering coefficients. One consequence of this approach is that it assumes that as the patterns of light propagate into the turbid medium, the spatial frequency remains constant. This is not true as defocus errors will broaden the effective area of the projected image as it propagates farther way from the ideal image plane, effectively reducing the spatial frequency. In this case, the measured broadening of the project pattern at a depth of 10 mm was an additional 2 mm, effectively decreasing the spatial frequency by <5% at its most extreme depth, even if all spatial frequencies were to reach that depth, that would result in a calculation error <5%.^21^ We acknowledge that this assumption is a limitation to this approach. Given its magnitude relative to the other aberration induced errors, however, we consider this residual error to be minimal.

Considerations to the optical design of the SFDI/S can also play a significant factor in these types of aberration induced errors. Rather than utilizing this empirical approach to compensate for these errors, careful lens design choices can minimize these aberrations and hence negate the potential need to utilize this method. The fitting equation [Eq. (1)] was derived empirically to describe the specific combinations of aberrations present in the system used in this investigation. It is expected that different systems would contain different combinations of aberrations and therefore may require another functional form to describe their depth-specific errors. Note that the terms in this equation do appear to correlate with chromatic aberration and depth of focus errors from a simple lens design. The first term in this equation is the most dominant and correlates with the expected behavior of chromatic aberration when only a single glass material is used in the optical system. If chromatically compensating lenses are used (e.g., achromats, apochromats, etc.), this simple power law term would no longer be sufficient as higher-order terms would be required to describe the increasingly complex chromatic dependence of focal length. The second term represents the slight wavelength dependent change in depth of focus due to the chromatic aberration shift in focal length and the last term represents the general depth of focus related to the numerical aperture of the imaging system.

From this investigation, the impact of the depth of field of the projected illumination has been shown to impact the recovery of subsurface and out-of-plane optical properties. There are several optical designs that can mitigate this type of aberration, e.g., high F/# systems, telecentric designs, or aspheric extended field approaches. These optical solutions, however, often come at a price in terms of practical clinical integration and use. Typically, these optical designs require long working distances and/or low light throughput that compete with clinical space and data acquisition times. It is for these reasons that this characterization and compensations methods are presented here as a pragmatic compromise on the instrumentation design while maintaining spectral integrity of the data collected.

## 5 Conclusion

We have provided a method to quantify primary optical aberrations' potential impact on spatial frequency domain measurements with respect to depth and proposed a simple empirical approach to compensate for these errors in post processing. We have shown that, even though these aberrations can considerably affect the spectral integrity of subsurface optical characteristics, they can be addressed independently from the turbid medium's intrinsic optical properties. This compensation method can be applied as an LUT to turbid media whose surface is displaced from the ideal focus as well as the subsurface tissue structures in layered media.
